# Prevalence and factors associated with Posttraumatic Stress Disorder among Healthcare Workers During the COVID-19 Pandemic: Findings from a survey applied on a Portuguese General Hospital

**DOI:** 10.1192/j.eurpsy.2023.882

**Published:** 2023-07-19

**Authors:** L. Paulino Ferreira, N. Ribeiro, M. Duarte

**Affiliations:** 1 Psychiatry and Mental Health, Centro Hospitalar de Setúbal, Setúbal; 2 Anatomy and Neuroanatomy, Nova Medical School | Universidade Nova Lisboa, Lisboa, Portugal

## Abstract

**Introduction:**

Posttraumatic Stress Disorder (PTSD) consists of an intense, prolonged, and occasionally delayed reaction to a deeply stressful event. PTSD is associated with risk of suicide and chronic psychological impairment. The continued exposure to stress suffered by Healthcare Workers (HCWs) during the COVID-19 pandemic can be considered a mass traumatic event and contribute to higher rates of PTSD in this population.

**Objectives:**

To study the rates of PTSD in a sample group of healthcare professionals working in a Portuguese general hospital and its relationship with a number of individual variables considered to be relevant by the existing literature on the subject.

**Methods:**

We devised a survey to assess the prevalence of PTSD among HCWs in a general hospital and its relationship with sex, social support, profession, work experience in healthcare, time spent caring for COVID-19 patients and place in which the COVID-19-related activities were carried out. PTSD symptoms were assessed using the PCL-5, Portuguese version.

**Results:**

A total of 226 HCWs were included in the study. Provisional diagnosis of PTSD was made based on the PCL-5 responses, considering DSM-5 criteria and the cutoff score of 33.

79 out of 226 (35.0%) HCWs had a provisional diagnosis of PTSD, and a significant association was found between PTSD and time spent working with COVID-19 patients and between PTSD and place of work, namely the COVID-19 Emergency Room and Intensive Care Unit.

**Conclusions:**

Our results are in line with previous studies, highlighting the importance of a serious, wide, and honest discussion about mental health promotion among HCWs. The COVID-19 pandemic has been an undeniable source of stress for HCWs around the globe, and the consequences of this stress are beginning to manifest themselves. It is urgent that this reflection takes place, as it is of paramount importance that what we all have lived in the past years serves as a lesson, and not as a warning of a crisis doomed to repeat itself.

**Disclosure of Interest:**

None Declared

